# Minor contribution of cytochrome P450 3A activity on fentanyl exposure in palliative care cancer patients

**DOI:** 10.1038/s41598-019-51279-6

**Published:** 2019-10-10

**Authors:** Marcus J. P. Geist, Victoria C. Ziesenitz, Hubert J. Bardenheuer, Juergen Burhenne, Gisela Skopp, Gerd Mikus

**Affiliations:** 1 Department of Anesthesiology, Heidelberg University Hospital, Heidelberg, Germany; 2 Department of Clinical Pharmacology and Pharmacoepidemiology, Heidelberg University Hospital, Heidelberg, Germany; 3 Department of Pediatric Cardiology, Heidelberg University Hospital, Heidelberg, Germany; 4 Forensic Toxicological Center, Munich, Germany; 5 Present Address: Palliative-Care-Team Leuchtturm, Groß-Gerau, Germany

**Keywords:** Quality of life, Chronic pain

## Abstract

Transdermal fentanyl is widely used to control pain in cancer patients. The high pharmacokinetic variability of fentanyl is assumed to be due to cytochrome P450 3A-mediated (CYP3A) N-dealkylation to norfentanyl in humans. However, recently published clinical studies question the importance of the described metabolic pathway. In this small study in palliative cancer patients under real-life clinical conditions, the influence of CYP3A on fentanyl variability was investigated. In addition to the determination of midazolam plasma concentration to reveal CYP3A activity, plasma concentrations of fentanyl and its metabolite, norfentanyl, were measured in identical blood samples of 20 patients who participated in an ongoing trial and had been on transdermal fentanyl. Fentanyl, norfentanyl, midazolam, and 1′-OH-midazolam were quantified by liquid chromatography/tandem mass spectrometry. Plasma concentrations of fentanyl and norfentanyl exhibited a large variability. Mean estimated total clearance of fentanyl and mean metabolic clearance of midazolam (as a marker of CYP3A activity) were 75.5 and 36.3 L/h. Both clearances showed a weak correlation and hence a minimal influence of CYP3A on fentanyl elimination.

## Introduction

Transdermal fentanyl is widely used as a basic therapy in the treatment of pain, which is one of the most common and distressing symptoms of cancer patients near the end of life^1^. Fentanyl is a synthetic full agonist at µ-receptors with high lipophilicity, submitting a sudden transfer of the agent to central nervous structures^2^. Cytochrome P450 3A (CYP3A) is the most relevant drug metabolizing enzyme in humans and responsible for metabolism of about half of all available pharmaceuticals. Fentanyl is assumed to be almost exclusively metabolized in the liver by CYP3A-mediated piperidine N-dealkylation to norfentanyl in humans^3,4^. The known interindividual high variability of CYP3A activity would result in a high variability of fentanyl exposure and in consequence also the efficacy and the side effects. Moreover, drug interactions are commonly caused by pharmaceuticals acting as an inducer or inhibitor of the CYP enzymes^5^. As drug interactions are often responsible for the escalation of debilitation symptoms, and therefore responsible for the negative influence of the palliative cancer patients’ quality of life, it is of major significance to be aware of a drug’s CYP-dependent metabolism.

As part of a clinical trial investigating the CYP3A activity in palliative cancer patients, this analysis is dealing with the metabolism of fentanyl in a special population under real-life clinical conditions in order to increase knowledge about the influence of CYP3A. The activity can be assessed in a subject by measuring a few plasma concentrations after oral intake of a microdose of midazolam, acting as a marker substance, in combination with pharmacokinetic calculations^6,7^. The determination of midazolam plasma concentrations as well as plasma concentrations of fentanyl and its metabolite norfentanyl in identical blood samples offers an exceptional opportunity of investigating the interindividual variability.

## Methods

The study was approved by the responsible ethics committee of the Medical Faculty at Heidelberg University (Heidelberg, Germany; approval number S-398/2015). Written informed consent was obtained from each patient prior to any study-related activity. This clinical trial was conducted according to the World Medical Association Declaration of Helsinki. It started in April 2016 at the palliative care unit of Heidelberg University Hospital and is still ongoing. The detailed study protocol of the trial was published before^8^.

Included patients were selected based on the administration of transdermal fentanyl to be evaluated in this study. Transdermal fentanyl was prescribed by the treating physicians due to a regular drug therapy and not modified by the investigators. Measuring plasma concentrations of substances other than midazolam was predefined in the study protocol and approved by the ethics committee. In addition to the determination of midazolam plasma concentration to reveal a four-hour pharmacokinetic profile after oral intake of a 10 µg microdose as part of the procedure of the main trial, plasma concentrations of fentanyl and its metabolite norfentanyl were measured in identical blood samples.

### Quantification of plasma concentrations

Fentanyl and norfentanyl have been measured by liquid chromatography/tandem mass spectrometry (LC-MS/MS) with electrospray source in positive ionization mode following protein precipitation. Briefly, 100 µl of serum was mixed with fentanyl-d5 (Cerilliant, Round Rock, Texas, USA) as the internal standard, and 1000 µl cold acetonitrile was added. Precipitated proteins were removed by centrifugation (10 min, 9000 g). The supernatant was reduced to dryness (under nitrogen, 37 °C) and reconstituted in 150 µl of the mobile phase. One µl was injected into the QTRAP 6500 LC-MS/MS-system (Sciex, Darmstadt, Germany). Fentanyl and norfentanyl were separated on a Zorbax Eclipse C8 column (150 × 4.6 mm, particle size 5 µm; Agilent, Waldbronn, Germany) by gradient elution (5 mM ammonium formate/0.1% formic acid; 5 mM ammonium formate in methanol/0.1% formic acid). Multiple reaction monitoring was used for specific detection of fentanyl m/z 337→188*, 337→105, norfentanyl m/z 233→84*, 233→55, and fentanyl-d5 m/z 342→105. Transitions marked with an asterisk were used for quantification. Calibration was performed by addition of standard solutions to drug-free matrix (0.1, 0.2, 0.3, 0.6, 1.0, 5.0 ng/ml; certified standards were purchased from Cerilliant, Round Rock, Texas, USA) prior to extraction; a blank and a zero sample as well as five quality control samples covering the calibration range were included in each run (n = 3, for all samples). Bench top stability was assured for both analytes up to 3 days. Linearity of calibration lines was assessed by regression analysis; correlations coefficients were 0.9994 and 0.9989 for fentanyl and norfentanyl, respectively. According to DIN 32645, the lower limit of detection and of quantitation were 0.02 and 0.05 ng/ml. The relative recovery from serum was on average 89% and 96% for fentanyl and norfentanyl, respectively (n = 6). Matrix effects were <25%. Percent mean within-day imprecision and accuracy of <4% and >93% for fentanyl and of <2% and >95% for norfentanyl were obtained (n = 8, respectively).

Plasma concentrations of midazolam and 1′-OH-midazolam were quantified using high-performance liquid chromatography with tandem mass spectrometric (LC/MS/MS) detection methods as previously described with lower limits of quantification of 0.093 pg mL^−1^ (midazolam) and 0.255 pg mL^−1^ (1′-OH-midazolam)^9^.

### Data analysis

Area under the plasma concentration-time curve (AUC) of midazolam was calculated between 2 and 4 hours after midazolam dosing (AUC_2-4_) using Prism® 7.0 (GraphPad Software, California, USA). Partial metabolic clearance of midazolam (CL_met_) was derived using the established equation CL_met_ = 5668/(AUC_2-4_/mg midazolam) as a reliable method to assess CYP3A activity^10,11^. In the same 4 samples, fentanyl and norfentanyl concentrations were determined. The samples for AUC_2-4_ of fentanyl were obtained at any times during this 72-hour dosing interval of the fentanyl patch. Assumptions were made for a constant delivery rate of fentanyl from the patch and also a constant resorption from the subcutaneous depot since the patients were hospitalized and in stable conditions. Therefore, total clearance (CL) of fentanyl was estimated using 2.78% of the dose of the fentanyl patch delivered for the whole 72-h dosing period divided by AUC_2-4_.

### Statistical analysis

Statistical analysis and graphic illustrations were performed using Prism® 7.0 (GraphPad Software, California, USA). Data are presented as mean ± SD.

## Results

Of the 41 patients included in the trial 20 patients were treated with transdermal fentanyl under real-life clinical conditions and thus assessed in this analysis. All of these patients suffered from cancer with impaired liver function based on the laboratory test results. According to the us presented previous findings and accessible patient’s charts none of the patients did receive cancer treatments due to their reduced general health condition at least 2 weeks before the day of the study. No special diet or nutritional supplements were applied. Patient characteristics including laboratory test results are described in Table 1.

**Table 1:** Patient characteristics.

Total patients	20 (100%)
Male/female	12 (60%)/8 (40%)
Age (years)	66 ± 12 (48–85)
BMI (kg/m²)	26.0 ± 8.8 (16.8–53.6)
Fentanyl patch dose (µg/h)	52 ± 51 (12.5–175)
**Primary tumor localization**	
Breast	2 (10%)
Larynx	2 (10%)
Ovary	3 (15%)
Pancreas	4 (20%)
Gastro intestinal tract	5 (25%)
Urinary tract	2 (10%)
Other	2 (10%)
**Laboratory test results (reference range)**	
Serum creatinine (0.5–1.0 mg/dL)	1.1 ± 0.6 (0.5–3.3)
Total bilirubin (<1.2 mg/dL)	1.7 ± 2.5 (0.2–9.3)
Aspartate transaminase (<50 IU/L)	116.5 ± 142.3 (14–572)
Alanine transaminase (<50 IU/L)	52.4 ± 44.5 (10–139)
Alkaline phosphatase (35–105 IU/L)	239.3 ± 169.7 (82–688)

Data presented as n (%) or mean ± SD (and range).

A dose increase due to the indication of the treating physicians was done in 2 patients at the last patch application before the pharmacokinetic assessment day. In 3 patients, administration of transdermal fentanyl was started at least 2 days earlier, and 18 patients were on constant fentanyl dosing for at least 1 dosing interval. As the evaluated subjects had been on individual patch doses ranging from 12.5 to 175 µg/h, all measured plasma concentrations were normalized to a dose of 25 µg/h transdermal fentanyl.

### Plasma concentrations

Assuming a constant sustained fentanyl transdermal delivery out of the patches under clinical conditions, plasma levels are anticipated to stay nearly constant. This is of importance as the blood samples were individually taken in relation to midazolam administration which translates to the following time points in relation to the fentanyl patch application: In 10, 5, 5 of the investigated patients the series of 4 blood samples for fentanyl analysis was taken at 2.8 (±1.56), 26.3 (±1.35), 50.3 (±1.35) hours after application of the patch, respectively.

**Figure 1:**
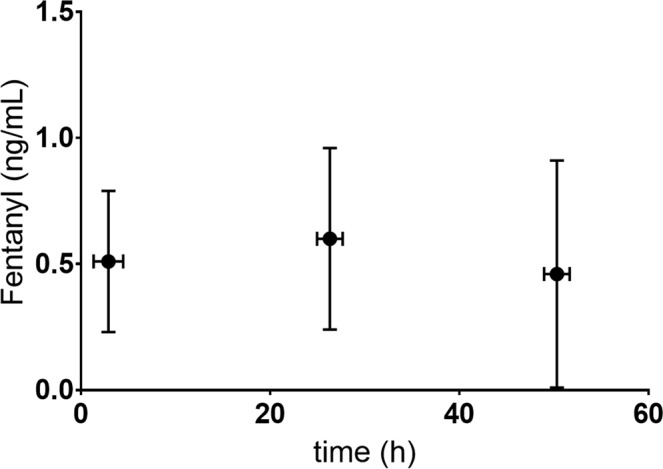
Plasma concentrations of fentanyl (ng/mL) and sampling time points in relation to fentanyl administration* (period between application of the patch and blood sampling). *Normalisd to 25 µg/h.

**Figure 2:**
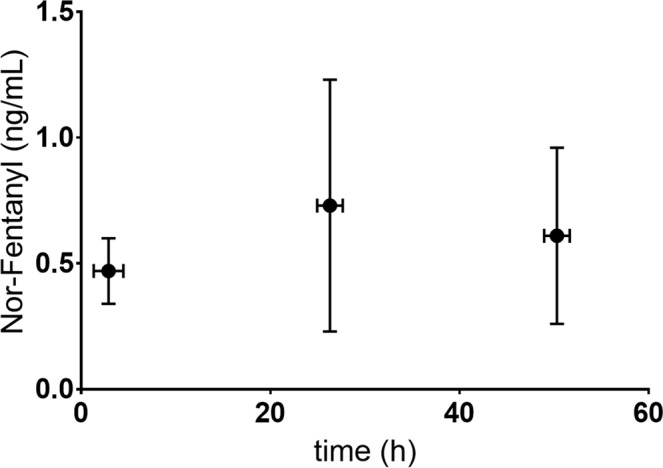
Plasma concentrations of norfentanyl (ng/mL) and sampling time points in relation to fentanyl administration* (period between application of the patch and blood sampling). *Normalised to 25 µg/h.

Mean dose normalized plasma concentrations of fentanyl and norfentanyl of all evaluated blood samples, in relation to the 3 mentioned mean sampling time points from fentanyl administration (period between application of the patch and blood sampling), are presented in Figs 1 and 2: Mean plasma concentrations of fentanyl and norfentanyl at the 3 time points during the dosing interval were rather similar on day 1, 2 or 3 after patch application, but the individual plasma concentrations exhibited a large variability. Lowest variability was observed when samples were taken a few hours after the new patch was applied.

**Figure 3:**
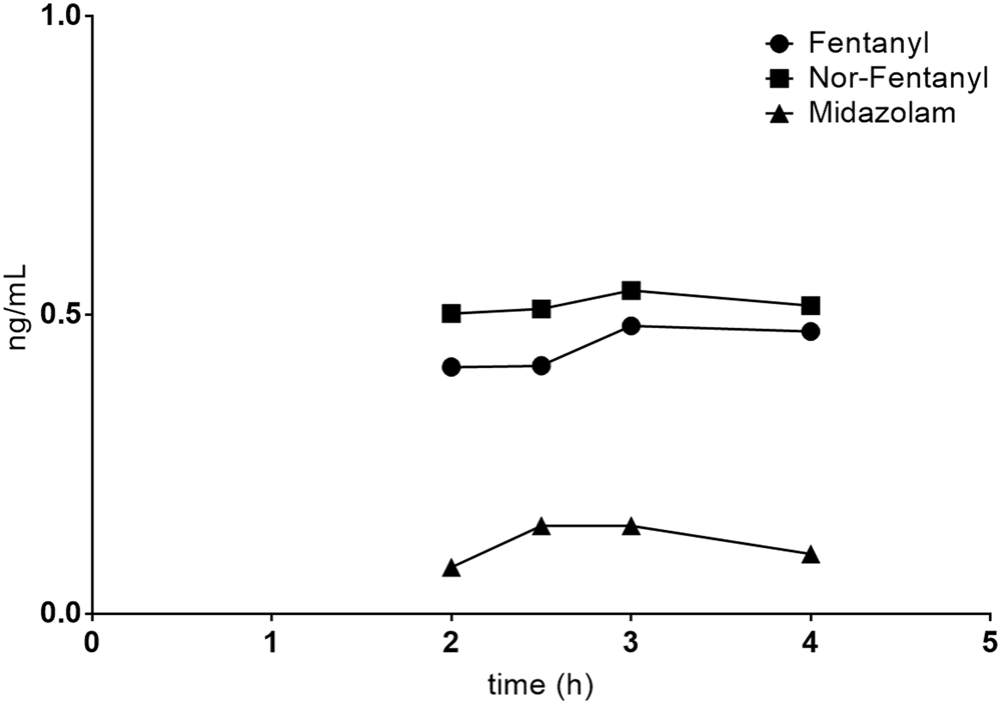
Mean plasma concentrations of fentanyl, norfentanyl and midazolam (ng/mL) in relation to midazolam administration.

Plasma concentrations of fentanyl, norfentanyl and midazolam in relation to midazolam administration are shown in Fig. 3.

### Pharmacokinetic and metabolism parameters

Pharmacokinetic data of fentanyl as well as midazolam are presented in Table 2. Compared to the metabolic ratio (MR) of norfentanyl and fentanyl (1.1 ± 1.3) the MR of 1′-OH-midazolam and midazolam was much higher (286 ± 236).

**Table 2:** Pharmacokinetic and metabolic parameters of fentanyl and midazolam presented as mean ± SD.

	Fentanyl transdermal*	Midazolam p.o.
Dose	12.5–175 µg/h	10 µg
AUC_2-4_ (pg*h/mL)	922 ± 555	85 ± 103
AUC_2-4_ (pg*h/mL) norfentanyl	1008 ± 705	
CL (L/h)	75.5 ± 44.7	
CL_met_ (L/h)		±31.3
MR**	1.1 ± 1.3	286 ± 236

^*^Normalized to 25 µg/h.

^**^Metabolic ratio (MR = AUC_2-4_ metabolite/AUC_2-4_ substrate).

**Figure 4:**
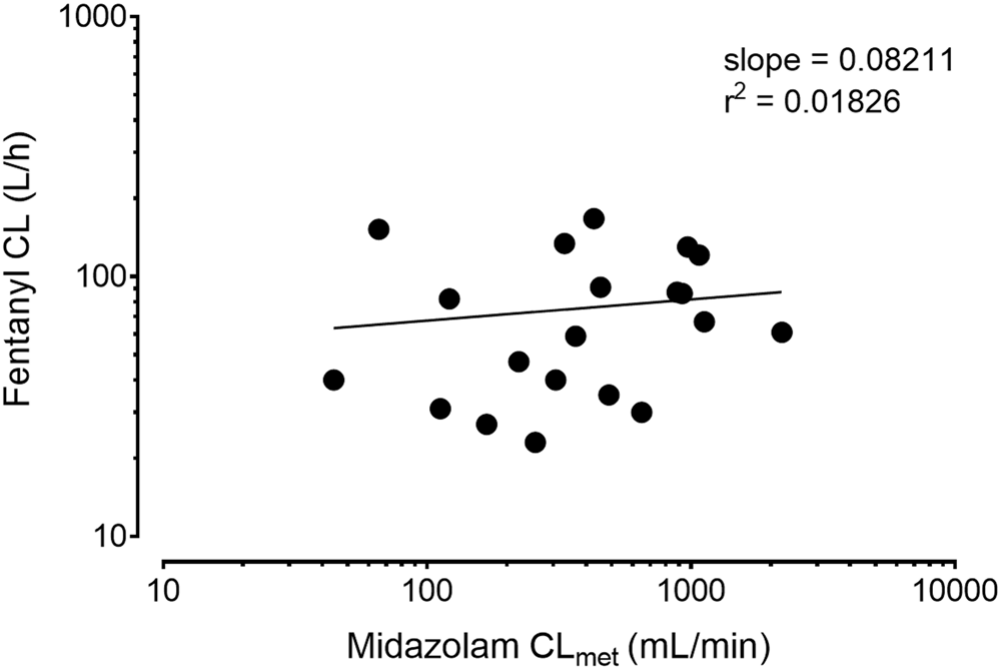
Correlation of fentanyl CL and midazolam CL_met_ in 20 patients. r^2^: coefficient of determination.

If metabolism of fentanyl to norfentanyl was mainly due to CYP3A, the total clearance of fentanyl should correlate with the metabolic clearance of midazolam to 1′-OH-midazolam, as the latter is known to be exclusively mediated by CYP3A. Hence, data of this evaluation were used to establish the correlation of fentanyl CL and midazolam CL_met_. Nonlinear regression analysis of the log transformed fentanyl CL and midazolam CL_met_ (Fig. 4) resulted in a weak relationship between the corresponding parameters, since the coefficient of determination is close to zero (r^2^ = 0.018). A slope of 0.08 reveals that about 8% of the total fentanyl clearance (fentanyl metabolism) is related to the midazolam metabolic clearance (marker of CYP3A activity).

## Discussion

This study investigated the variability of exposure of transdermal fentanyl in palliative cancer patients under real-life clinical conditions. Evaluated patients exhibited a large variability in fentanyl plasma concentrations, which is of importance in regards to effectiveness and safety of the transdermal formulation. These findings are in line with a work by Barratt *et al*. who identified substantial interindividual variabilities of fentanyl serum concentrations as well as of the corresponding metabolic ratio among 620 cancer patients on transdermal fentanyl. CYP3A gene polymorphisms can influence transdermal fentanyl metabolism to norfentanyl, but only a small proportion of the variability of serum fentanyl and norfentanyl concentrations can be explained by these polymorphisms. An unexplained large interindividual variability of fentanyl serum concentrations with potential clinical consequences and metabolism of fentanyl to norfentanyl was observed and is still awaiting further research to determine the underlying mechanisms^12^.

**Figure 5:**
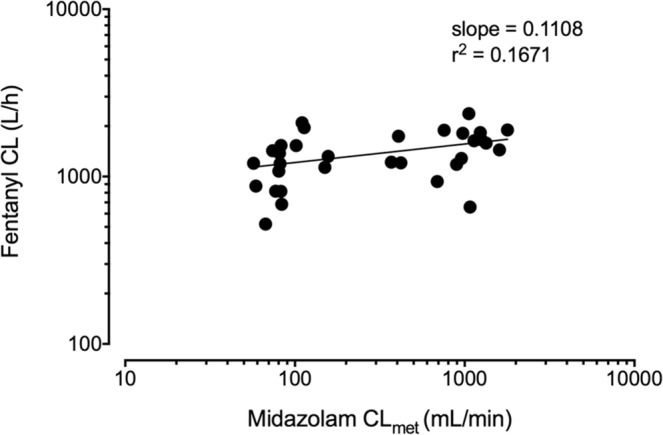
Correlation of fentanyl CL and midazolam CL_met_ in 16 healthy volunteers. r^2^: coefficient of determination.

Among the expert prescribing information of fentanyl, an almost exclusively CYP3A-dependent metabolism is reported by several studies, which implicates a high chance of drug interactions if fentanyl is given together with other substances. Thus the clearance of intravenously administered fentanyl was significantly reduced (by −23% and −16%) by voriconazole and fluconazole (both CYP3A inhibitors) in healthy volunteers. However, AUC ratios between norfentanyl and fentanyl were reduced more than the corresponding clearances, which led to the suggestion that other pathways may be involved in the metabolism of fentanyl^13^. This hypothesis is powered by findings of other groups, that revealed inconsistencies in respect to the metabolism of fentanyl. Concomitant administration of transdermal fentanyl and aprepitant, another CYP3A inhibitor that is extensively metabolized by the enzyme, showed no significant influence on the AUC of fentanyl as it was expected before^14^. However, norfentanyl was not quantified in that study. Furthermore, the total clearance of intravenous fentanyl was inhibited just weakly though a strong inhibition of the metabolic clearance of fentanyl to norfentanyl was observed to be affected by the antimycotic and strong CYP3A inhibitor ketoconazole in healthy volunteers^15^. In addition, we re-analysed data from that study in which fentanyl was given intravenously alone and during CYP3A inhibition by ketoconazole, and midazolam was co-administered orally as CYP3A probe drug. This new correlation analysis of the clearances of fentanyl and midazolam under baseline and inhibition conditions results in a weak relationship (Fig. 5). Indeed, the slope was 0.111, which is very similar to the slope in our patients (0.08) indicating only a minor contribution of CYP3A to the fentanyl clearance. The range of the midazolam clearances in both the patients and the healthy volunteers (under baseline and inhibition) is very similar, allowing for a meaningful comparison between both study groups. Additional studies with only small effects of CYP3A inhibitors on fentanyl exposure compared to other substrates of the enzyme are summarized by Kuip *et al*.^16^.

Although the use of a limited sampling strategy to estimate midazolam metabolic clearance has been validated, this method has not been proven for fentanyl, which could be a limitation of our study. Furthermore, a constant fentanyl delivery from the patch during the 72-hour dosing interval into the subcutaneous depot was assumed, however, a constant resorption from the depot into the systemic circulation cannot be assured. Regarding the real-life clinical setting of the trial, an intravenous fentanyl application with secured constant systemic drug application was not realizable in the investigated palliative care patients. However, the correlation of the CYP3A dependent activities of the two substances in identical blood samples illustrates a valuable approach to understanding the variability of transdermal fentanyl effect under clinical conditions.

## Conclusion

The present study did not confirm the widely accepted assumption that the main route of fentanyl elimination is via CYP3A-mediated N-dealkylation to norfentanyl. The results obtained in this special population, under real-life clinical conditions, supports the recent data obtained in healthy volunteers^15^. According to our findings, this pathway is only responsible for about 8% of the total metabolism of the drug, and hence is not the major factor for the high variability of fentanyl pharmacokinetics. Based on this current observation, no major drug interactions are to be expected even when strong CYP3A inhibitors are concomitantly used.

## Data Availability

The datasets generated during and analysed during the current study are available from the corresponding author on reasonable request.
